# Determinants of quality of life among electrical technicians

**DOI:** 10.1192/j.eurpsy.2026.11816

**Published:** 2026-07-31

**Authors:** I. Sellami, A. Abbes, A. Feki, M. L. Masmoudi, M. Hajjaji, K. Jmal Hammami

**Affiliations:** 1 occupational medicine; 2 Rheumatoloy, Hedi Chaker Hospital, sfax, Tunisia

## Abstract

**Introduction:**

Enhancing workers’ quality of life is a key factor in improving productivity. This quality of life is shaped by various occupational determinants, including working conditions and psychosocial factors, as well as mental health, all of which warrant in-depth investigation.

**Objectives:**

This study aimed to identify the determinants of quality of life among electrical technicians.

**Methods:**

We conducted a descriptive, analytical and cross-sectional survey among electrical technicians. Data collection was carried out using a self-completed questionnaire. We collected socio-professional data. The job satisfaction was evaluated using the single-item measure of job satisfaction. To assess the perceived workload, we choose the National Aeronautics and Space Administration Task Load Index (NASA-TLX). In this study, we evaluated raw NASA-TLX scores. We assessed the quality of life of workers by the questionnaire three-level EuroQol EQ-5D-3L. We used utility values for EQ-5D-3L for the Tunisian population.

**Results:**

Our study population was exclusively male, including 70 electrical technicians. The mean age of participants was 38.1 ±10.2 years. The mean of the tenure of job was 14.5±11.1 years. Median job satisfaction was 4, with extreme values ranging from 1 to 5. The mean score of mental demand, physical demand, performance, effort, frustration level and temporal demand were respectively 88.7±14.4, 62.3±22.7, 85.1±13.8, 82.7±14.3, 36.9±29.3 and 59.8±28.6. The mean of EQ-5D-5L index value was 0.93±0.1. The quality of life of workers was associated to higher job satisfaction (p=0.01, r= 0.29) and lower frustration level (p <0.001, r = -0.4).

**Conclusions:**

Workers’ quality of life depends on their job satisfaction and mental health. Improving working conditions contributes to enhancing this quality of life and, consequently, their productivity.

**Disclosure of Interest:**

None Declared

